# Association between blood volatile organic aromatic compound concentrations and hearing loss in US adults

**DOI:** 10.1186/s12889-024-18065-0

**Published:** 2024-02-27

**Authors:** Senlin Wang, Jing Luo, Fang Zhang, Ruimin Zhang, Wantao Ju, Nianwei Wu, Jianhui Zhang, Yanjun Liu

**Affiliations:** 1 College of Medicine, Southwest Jiaotong University, Chengdu, China; 2 The Center of Gastrointestinal and Minimally Invasive Surgery, Department of General Surgery, The Third People’s Hospital of Chengdu, The Affiliated Hospital of Southwest Jiaotong University, Chengdu, China; 3 Department of Otolaryngology head and neck surgery, The Third People’s Hospital of Chengdu, Affiliated Hospital of Southwest Jiaotong University, Chengdu, Sichuan 610031 China; 4 School of Life Science and Engineering, Southwest Jiaotong University, Chengdu, China; 5 Department of General Surgery, Center for Obesity and Metabolic Health, The Third People’s Hospital of Chengdu, The Affiliated Hospital of Southwest Jiaotong University, Chengdu, China; 6 Research Center for Obesity and Metabolic Health, College of Medicine, Southwest Jiaotong University, Chengdu, China; 7 Medical Research Center, The Third People’s Hospital of Chengdu, The Affiliated Hospital of Southwest Jiaotong University, Chengdu, China

**Keywords:** Volatile organic aromatic compound concentrations, Hearing loss, NHANES, Epidemiology

## Abstract

**Objective:**

Benzene, ethylbenzene, meta/para-xylene, and ortho-xylene, collectively referred to as benzene, ethylbenzene, and xylene (BEX), constitute the main components of volatile organic aromatic compounds (VOACs) and can have adverse effects on human health. The relationship between exposure to BEX and hearing loss (HL) in the adult U.S. population was aimed to be assessed.

**Methods:**

Cross-sectional data from the National Health and Nutrition Examination Survey (NHANES) for the years 2003–2004, 2011–2012, and 2015–2016 were analyzed. This dataset included complete demographic characteristics, pure-tone audiometry measurements, and volatile organic compound detection data from the NHANES database. A weighted multivariate logistic regression model was employed to investigate the associations between blood BEX concentrations HL, low-frequency hearing loss (SFHL), and high-frequency hearing loss (HFHL).

**Results:**

2174 participants were included, with weighted prevalence rates of HL, SFHL, and HFHL being 46.81%, 25.23%, and 45.86%, respectively. Exposure to benzene, ethylbenzene, meta/para-xylene, and ortho-xylene, and cumulative BEX concentrations increased the risk of hearing loss (odds ratios [ORs] were 1.36, 1.22, 1.42, 1.23, and 1.31, respectively; all *P* < 0.05). In the analysis with SFHL as the outcome, ethylbenzene, m-/p-xylene, o-xylene, benzene, and overall BEX increased the risk (OR 1.26, 1.21, 1.28, 1.20, and 1.25, respectively; all *P* < 0.05). For HFHL, exposure to ethylbenzene, m-/p-xylene, o-xylene, benzene, and overall BEX increased the risk (OR 1.36, 1.22, 1.42, 1.22, and 1.31, respectively; all *P* < 0.05).

**Conclusion:**

Our study indicated that a positive correlation between individual or cumulative exposure to benzene, ethylbenzene, meta/para-xylene, and ortho-xylene and the risk of HL, SFHL, and HFHL. Further research is imperative to acquire a more comprehensive understanding of the mechanisms by which organic compounds, notably BEX, in causing hearing loss and to validate these findings in longitudinal environmental studies.

**Supplementary Information:**

The online version contains supplementary material available at 10.1186/s12889-024-18065-0.

## Introduction

According to the 2021 World Health Organization’s World Hearing Report, nearly one-quarter of the global population will experience varying degrees of hearing loss by 2025, resulting in an estimated economic loss of 980 billion USD [1]. This financial burden is accompanied by increased risks of conditions like Alzheimer’s disease and dementia in individuals with age-related hearing loss [2, 3]. Additionally, individuals with hearing loss often face communication difficulties that lead to linguistic degradation, reduced social engagement, social isolation, emotional detachment, and depression [4, 5]. Among the factors contributing to hearing loss, ototoxic mechanisms have been a central focus, with clinical research primarily centered around the adverse effects of platinum-based drugs and aminoglycosides [6, 7]. Some pioneers have investigated the effects of trace heavy metals (such as lead and cadmium), ingested and accumulated by humans, on hearing loss, revealing the stress-induced damage and toxic effects of these metals on cochlear hair cells [8–10]. However relationship between accumulated environmental pollutants in human blood and hearing loss has not yet been thoroughly researched.

Benzene, ethylbenzene, meta/para-xylene (m-/p-xylene), and ortho-xylene (o-xylene) (collectively referred to as Benzene, Ethylbenzene, and Xylene or BEX) are aromatic compounds containing benzene rings that exhibit strong volatility at room temperature, making them prominent pollutants within the group of volatile organic aromatic compounds (VOACs). BEX originates from various sources, primarily including gas emissions resulting from the combustion of petroleum products, chemical solvents, paints, and other building materials. BEX is recognized as a carcinogen by the International Agency for Research on Cancer, and prolonged exposure to BEX has been demonstrated to disrupt reproductive function, induce asthma, and lead to leukemia [11–13]. Accumulation of BEX in the human body can occur through inhalation, ingestion of contaminated water sources, and subsequent dissolution in the bloodstream, thereby spreading to various organs and tissues. This has raised public concerns regarding the potential life and health threats posed by BEX. In recent years, BEX has been implicated in the discovery of additional potential health issues. For instance, benzene poisoning has been linked to immune suppression and splenic damage through the B-cell receptor signaling pathway [14]. Partha et al. demonstrated through epidemiological research that BEX exposure contributes to premature birth in pregnant women [15]. 

Observational studies also have found a positive correlation between the occupational workers’ urinary biomarkers of toluene, such as hippuric acid and ortho-cresol, and toxicity to the auditory system [16, 17]. Wenzhen Li et al. pointed out that the concentration of polycyclic aromatic hydrocarbons (PAHs) in urine is related to various frequencies of hearing loss across different age groups [18]. However, most solvents have a short biological half-life, and varying degrees of metabolism, and a small amount of the solvent is excreted in the urine in metabolized forms [19]. Therefore, using organic solvents in urine as biomarkers for hearing loss has certain limitations. Considering this limitation, the focus of this study has shifted towards a comprehensive exploration of the relationship between blood BEX concentrations and hearing loss in the general population. Through this research, we hope to provide a more comprehensive understanding of the association between environmental factors and auditory health.

## Methods

### Study population

Given that the National Health and Nutrition Examination Survey (NHANES)working group collected auditory data from adults in the following three cycles: 2003–2004, 2011–2012, and 2015–2016, our study incorporates the aforementioned three cycles for investigation. We excluded participants aged < 20 years (*n* = 13,529) and participants aged > 60 years (*n* = 5250). In this study, we initially recruited participants for the measurement of benzene, ethylbenzene, o-xylene, and m/p-xylene, resulting in the exclusion of participants with missing data for these four volatile organic compound concentrations (VOACs) (*n* = 948). Participants with missing data for family income-poverty ratio (*n* = 189), lack of marital status (*n* = 2), self-reported cerumen or collapsing external ear canals (*n* = 612), wearing hearing aids (*n* = 17), and suffering from Parkinson’s (*n* = 13) were further excluded.

According to previous studies [20], NHANES recorded objective indicators of Eustachian Tube Dysfunction (ETD), specifically the middle ear pressures in participants’ left and right ears (measured in daPa units). Based on the standard tympanogram classifications by Linden11 and Jerger1, we defined peak middle ear pressures below 99 daPa as type C, middle ear pressures with compliance values of 0.2 as type B, and all other middle ear pressures as type A. If a participant had tympanometric type B or C in either or both ears, they were categorized as abnormal and excluded from the analysis (*n* = 264).

To exclude the possibility of drug-induced hearing loss, we extracted information on participants’ prescription medication use from the Prescription Medication Section (DSQ) of NHANES. Upon matching, the types of medications taken by participants were primarily focused on treating conditions such as diabetes, hypertension, infections, and hyperlipidemia, among others. As detailed information regarding participants’ medication, including dosage, duration, administration method, and brand was unavailable, we relied on clinical drug experience and existing literature evidence to primarily define participants taking the following 11 drugs (acetaminophen [21], hydrocodone [22], ciprofloxacin [23], phenytoin [24], levofloxacin [25], rifampin [26], minocycline [27], aspirin [28], metronidazole [29], nitroglycerin [30], and bumetanide [31]) as a high-risk group for drug-induced hearing loss and subsequently excluded them from the study (*n* = 46).

**Fig. 1:**
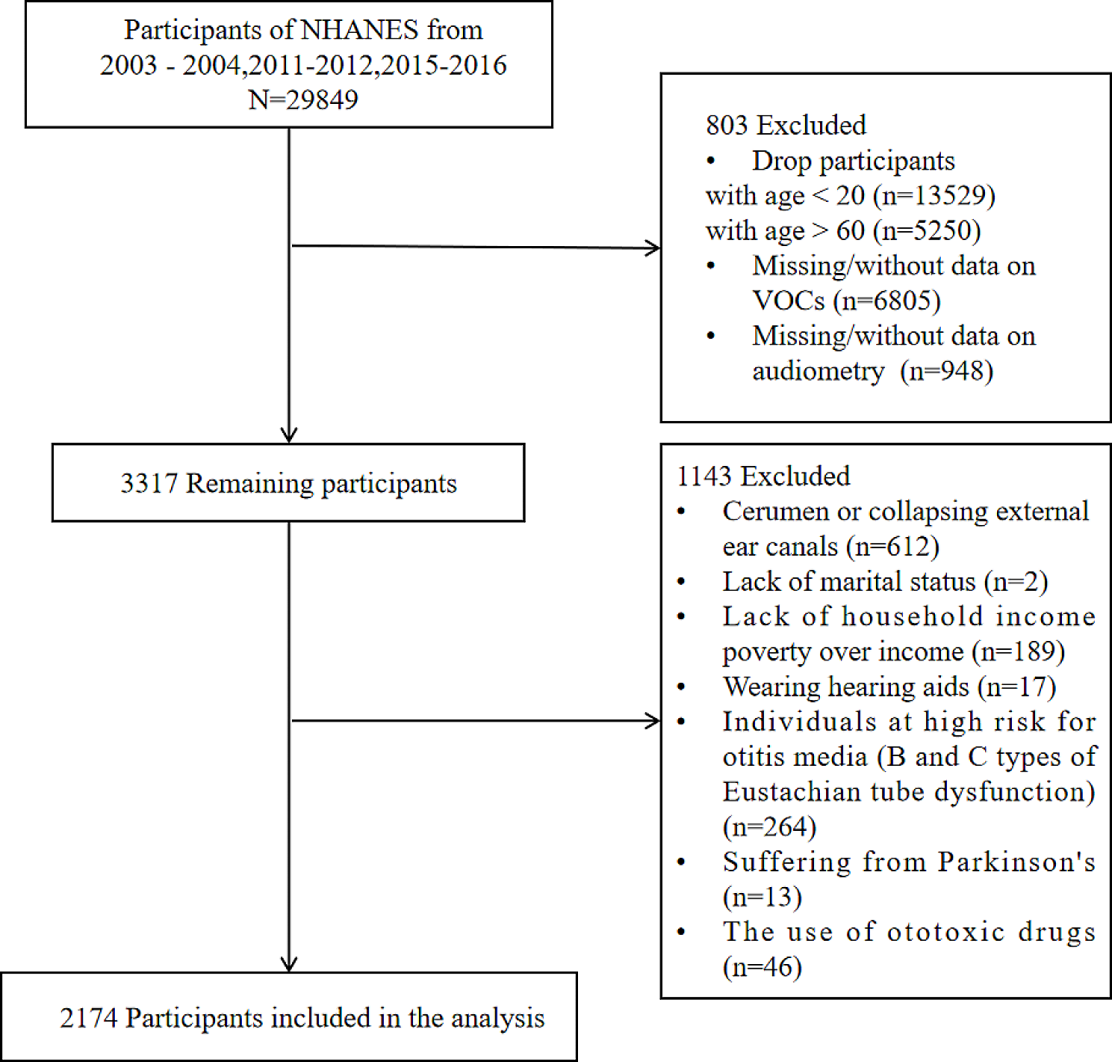
Flow chart of the patients included in the study

Ultimately, a total of 2174 participants were included for analysis (Fig. 1). The NHANES study protocol was approved by the Institutional Review Board of the National Center for Health Statistics. All participants provided informed consent.

### Blood aromatic volatile organic compounds determination

Participants arrived at a central location consisting of Mobile Examination Centers (MEC), where blood samples were collected. Analysis of the blood samples was performed using automated methods employing capillary gas chromatography (GC) and mass spectrometry (MS) combined with selected ion monitoring (SIM) detection and isotope dilution techniques. This analytical method was established to quantify VOACs in the blood of non-occupationally exposed individuals within this range, making it suitable for identifying cases with these levels of exposure over a sustained or recent period of low-level exposure.

### Audiometric measures and hearing loss

All sections of the audiometry exam were conducted by trained examiners on participants in a dedicated sound-isolating room at the MEC. Hearing threshold testing was performed on both ears of participants at seven frequencies: 500, 1000, 2000, 3000, 4000, 6000, and 8000 Hz. Hearing loss was characterized by pure-tone averages surpassing 25 dB in both ears among adults. Individuals were classified as having hearing loss if their hearing thresholds exceeded 25 dB in either ear across any of the examined frequencies. (500, 1000, 2000, 3000, 4000, 6000, and 8000 Hz) [32, 33]. 

Moreover, the research delved into the examination of two distinct categories of auditory impairment, encompassing Speech Frequency Hearing Loss (SFHL) and High-Frequency Hearing Loss (HFHL), which do not exhibit a mutually exclusive relationship. The assessments for SFHL were conducted at frequencies of 500, 1000, 2000, and 4000 Hz, while the evaluations for HFHL were performed at 3000, 4000, 6000, and 8000 Hz [34, 35]. 

### Ascertainment of covariates

Demographic particulars, encompassing age, sex, racial/ethnic background, and marital status, were garnered through a universally recognized questionnaire. Height, gauged in meters, and weight, measured in kilograms, were acquired following a stipulated protocol, subsequently utilized in the computation of body mass index (BMI), denoted in kilograms per square meter. Then the participants could be further separated into three subgroups, normal weight (BMI < 25 kg/m^2^), overweight (25 kg/m^2^ ≤ BMI < 30 kg/m^2^), and obese (BMI ≥ 30 kg/m^2^) [36]. Education level was categorized as below high school, high school or higher, and college or higher. Smoking status was classified as non-smoking (less than 100 lifetime cigarettes) and smoking (more than 100-lifetime cigarettes) [10]. Drinking status were categorized into two groups: those who consumed alcohol (a minimum of 12 beverages annually) and those who abstained from alcohol [37]. Hypertension was defined as self-reported physician diagnosis or current use of antihypertensive medication. Diabetes was defined as self-reported physician diagnosis or current use of antihyperglycemic medication. Ascertaining household income levels was accomplished through the utilization of the self-reported family income-poverty ratio (PIR), which was subsequently categorized into three distinct groups (0–1.3, 1.3–3.5, and > 3.5) [38]. The participant selection process was conducted in the aforementioned order.

In the NHANES cycles of 2003–2004, the questionnaire design related to hearing tests differs from the surveys conducted in 2011–2012 and 2015–2016. In the absence of questions regarding occupational noise exposure in the hearing questionnaire of the 2003–2004 cycle, according to the NHANES guidance manual, information on occupational noise exposure was obtained from the Occupation Section of the SP Household Questionnaire (OCQ). Therefore, in our study, occupational noise exposure is defined as “currently exposed to loud noise at work for an average of ≥ 4 hours/day” or “presence of noisy work conditions: ever exposed, 3 months or more.” Firearm noise exposure is defined as “presence of firearm noise exposure outside of work.” Recreational noise exposure is defined as “presence of other types of noise exposure, such as loud music, outside of work” [39]. 

For participants in the 2011–2012 and 2015–2016 cycles, occupational noise exposure is defined as “ever exposed to noisy work conditions for 4 hours or longer, several days a week.” Firearm noise exposure is defined as “use of firearms for any reason.” Recreational noise exposure is defined as “exposure to very loud noise or music for 10 hours or longer per week outside of work” [40]. 

### Statistical analysis

NHANES formulates survey-specific weighting factors to accommodate intricate survey structure, non-participation, and post-stratification, thereby guaranteeing that the derived estimations accurately portray the demographic composition of the non-institutionalized civilian populace in the United States. Regarding the measurement of volatile organic compounds in blood, BEX’s data are weighted using the corresponding subsample weight (WTSVOC2Y) for each cycle. NHANES data is collected in cycles of two years each, and since our data spans three cycles, the weight for each cycle is calculated as 2/6 * WTSVOC2Y to account for the proportional representation of each cycle. The statistical analysis was performed according to the guidelines provided in the NHANES data documentation [41]. 

Due to the skewed distribution of blood VOACs data, we performed a logarithmic transformation of BEX for our analysis. When describing the baseline characteristics of the study population, the data are presented as weighted means ± standard errors (SD) for continuous measurements, and as unweighted counts along with weighted percentages for categorical measurements. For continuous variables, statistical significance was assessed using Student’s *t* test. Similarly, for categorical variables, *P*-values were determined through the utilization of chi-square tests.

Weighted logistic regression, we addressed the intricacies of the survey design elements within the statistical analysis. To comprehend the potential confounding influences of diverse covariates, we systematically introduced adjustments across two successive models. Model 1 adjusted for age (continuous years), BMI, sex, race (non-Hispanic white, non-Hispanic black, Mexican American, etc.). Model 2 adjusted for variables in Model 1 plus education level (below high school, high school or equivalent, college or higher), marital status (married, separated including widowed and divorced groups, unmarried), smoking status (no, yes), drinking status (no, yes), hypertension (no, yes), diabetes (no, yes), and PIR levels (0–1.3, 1.3–3.5, and > 3.5).

In order to assess the robustness of the identified associations, we conducted three sensitivity analyses. Firstly, in the latest edition (2021) of “The World Report on Hearing” by the WHO), new recommendations for hearing classification have been proposed, introducing updated criteria for grading hearing loss [42]. Accordingly, we have reclassified participants into three levels based on the average hearing threshold (PTA): normal (< 20 dB), mild (20–35 dB), and moderate to severe (> 35 dB). Subsequently, we employed weighted ordered logistic regression analysis to investigate the relationship between BEX and varying degrees of hearing loss. Secondly, to mitigate the impact of age, participants aged > 40 were excluded, and a weighted logistic regression analysis was subsequently conducted. Lastly, given that one of the significant sources of VOCs is cigarette smoke, and previous studies have identified environmental tobacco smoke (ETS) exposure as a risk factor for hearing loss [43], we extracted biochemical test results for ‘serum cotinine’ from NHANES. Serum cotinine serves as a biomarker to quantify tobacco smoke exposure, and individuals with serum cotinine > 0.015 ng/mL are considered acutely exposed to ETS [44]. This variable was included as a control in the model for weighted logistic regression analysis.

As part of our analysis, we conducted subgroup analyses based on gender (male and female), age (20–40 years and 40–60 years), BMI categories (< 25, 25-29.9, and ≥ 30 kg/m^2^), smoking status (no and yes), diabetes (no and yes), hypertension (no and yes), education levels (below high school, high school or higher, and college or higher), and PIR (0–1.3, 1.3–3.5, and > 3.5) were subjects of investigation. Furthermore, potential effect variations were explored by introducing a product term for each stratifying variable and BEX into the primary model, followed by evaluation through a Wald test.

The dose-response relationship between VOACs and the occurrence of hearing loss was examined using Restricted Cubic Splines with three knots positioned at the 1th, 50th, and 90th percentiles. Advanced statistical analyses were carried out utilizing STATA version 17.0 and R version 4.3.1. Significance was determined when the two-tailed *P*-value fell below 0.05.

## Results

### Basic characteristics

Over the span of three NHANES cycles, encompassing the years 2003–2004, 2011–2012, and 2015–2016, a cumulative participant cohort of 29,849 individuals was included. After eliminating individuals below the age of 20 and those surpassing 60 years, lack of blood organics measurement information and hearing measurement information, cerumen embolism and collapse of the external auditory canal, lack of marriage information, and insufficient household income, 2174 participants were finally included in the analysis. The participants exhibited an average age of 39.20 years. Among the participants, there were a total of 995 instances of hearing loss, with 513 cases categorized as SFHL and 973 cases as HFHL. Additionally, 491 participants demonstrated the concurrent occurrence of two distinct types of hearing loss. The sample included in this study is representative of a weighted population of 77,433,612 noninstitutionalized U.S. adults.

Table 1 presents the characteristics of participants based on the presence or absence of hearing loss, SFHL, and HFHL. In our study population, the weighted prevalence of hearing loss was 46.81%, low-frequency hearing loss was 25.23%, and high-frequency hearing loss was 45.86%. Apart from race, alcohol drinking history, exposure to recreational noise, exposure to firearm noise, and PIR, no significant differences were observed among the groups in terms of these characteristics. However, statistically significant differences were noted in other baseline characteristics. Compared to participants without hearing loss, those with higher age, BMI, male, education level of High school or equivalent, marital status, history of smoking, alcohol consumption, hypertension, exposure to work noise, and diabetes had a higher prevalence of hearing loss. These associations remained consistent for both low-frequency and high-frequency hearing loss. Detailed characteristics of the study population are provided in Supplementary Tables 1–2.

**Table 1:** Baseline characteristics of study participants in NHANES 2003–2004,2011–2012 and 2015–2016 according to hearing loss

Characteristics		Non-hearing loss	Hearing loss			SFHL		HFHL	
		(*N* = 1179)	(*N* = 995)	*P-value		(*N* = 513)	*P-value	(*N* = 973)	*P-value
**Age, Mean (SD)**		34.24 (9.87)	44.84 (10.66)	< 0.001		46.83 (10.00)	< 0.001	44.83 (10.66)	< 0.001
**BMI, Mean (SD)**		28.43 (7.14)	29.67 (6.47)	0.004		29.80 (6.05)	0.036	29.69 (6.48)	0.004
**Gender, n, %**				< 0.001			< 0.001		< 0.001
Male		497 (42.7%)	542 (54.6%)			347 (67.1%)		536 (55.1%)	
Female		682 (57.3%)	453 (45.4%)			166 (32.9%)		437 (44.9%)	
**Race, n, %**				0.077			0.012		0.117
Mexican American		168 (8.9%)	151 (8.1%)			80 (8.0%)		146 (8.0%)	
Other Hispanic		108 (6.6%)	109 (6.2%)			52 (5.1%)		107 (6.2%)	
Non-Hispanic White		468 (65.4%)	413 (69.9%)			233 (73.3%)		404 (70.0%)	
Non-Hispanic Black		251 (11.5%)	165 (8.0%)			73 (6.2%)		164 (8.1%)	
Other Race - Including Multi-Racial		184 (7.6%)	157 (7.8%)			75 (7.4%)		152 (7.7%)	
**Education levels, n, %**				< 0.001			< 0.001		< 0.001
Less than high school		158 (9.1%)	229 (15.2%)			130 (16.6%)		226 (15.3%)	
High school or equivalent		611 (50.4)	521 (53.8%)			278 (57.1%)		508 (53.5%)	
College or above		410 (40.5%)	245 (31.0%)			105 (26.3%)		239 (31.2%)	
**Marital status, n, %**				< 0.001			< 0.001		< 0.001
Married		539 (47.9%)	568 (59.4%)			298 (59.9%)		559 (59.8%)	
Widowed		4 (0.4%)	20 (1.6%)			9 (1.6%)		19 (1.4%)	
Divorced		61 (4.5%)	112 (12.0%)			60 (12.5%)		109 (12.0%)	
Separated		36 (2.1%)	41 (3.0%)			22 (3.2%)		38 (2.8%)	
Never married		375 (31.5%)	162 (16.0%)			73 (14.4%)		159 (16.1%)	
Living with partner		164 (13.6%)	92 (8.0%)			51 (8.4%)		89 (7.9%)	
**Smoking status, n, %**				< 0.001			< 0.001		< 0.001
NO		761 (62.9%)	521 (50.6%)			229 (44.5%)		506 (50.6%)	
YES		418 (37.1%)	474 (49.4%)			284 (55.5%)		467 (49.4%)	
**Drinking status, n, %**				0.038			0.018		0.055
NO		859 (77.4%)	653 (72.2%)			334 (69.8%)		640 (72.3%)	
YES		320 (22.6%)	342 (27.8%)			179 (30.2%)		333 (27.7%)	
**Hypertension, n, %**				< 0.001			< 0.001		< 0.001
NO		982 (84.8%)	708 (73.0%)			348 (68.6%)		689 (72.7%)	
YES		197 (15.2%)	287 (27.0%)			165 (31.4%)		284 (27.3%)	
**Diabetes, n, %**				< 0.001			< 0.001		< 0.001
NO		1130 (96.3%)	879 (91.2%)			446 (89.3%)		857 (81.1%)	
YES		49 (3.7%)	116 (8.8%)			67 (10.7%)		116 (8.9%)	
**PIR, n, %**				0.640			0.768		0.604
<1.3		336 (21.8%)	293 (19.9%)			158 (20.0%)		287 (18.9%)	
1.3–3.5		438 (35.2%)	379 (36.1%)			199 (37.0%)		367 (35.6%)	
≥3.5		405 (43.0%)	323 (44.0%)			156 (43.0%)		319 (45.5%)	
**Noise exposure, yes, n, %**									
Work noise		141 (12.4%)	177 (18.3%)	0.009		110 (21.9%)	< 0.001	338 (18.3%)	0.759
Recreational noise		184 (15.8%)	177 (19.0%)	0.169		109 (22.2%)	0.012	175 (19.1%)	0.161
Firearm noise		402 (42.7%)	343 (42.4%)	0.901		205 (47.5%)	0.014	173 (43.0%)	0.015
**Year cycle, n, %**				0.899			0.678		0.931
2003–2004		223 (19.7%)	293 (20.3%)			99 (21.5%)		170 (19.7%)	
2011–2012		388 (33.8%)	379 (34.6%)			166 (32.6%)		330 (34.8%)	
2015–2016		568 (46.5%)	323 (45.1%)			248 (45.9%)		473 (45.5%)	
**Log (ethylbenzene), Mean (SD)**		-3.72 (0.61)	-3.49 (0.83)	< 0.001		-3.43 (0.77)	< 0.001	-3.49 (0.83)	< 0.001
**Log (m-/p-Xylene), Mean (SD)**		-2.71 (0.83)	-2.46 (0.99)	< 0.001		-2.37 (0.93)	< 0.001	-2.46 (0.99)	< 0.001
**Log(o-Xylene), Mean (SD)**		-3.73 (0.53)	-3.55 (0.70)	< 0.001		-3.51 (0.64)	< 0.001	-3.55 (0.70)	< 0.001
**Log (benzene), Mean (SD)**		-3.56 (0.82)	-3.31 (1.07)	< 0.001		-3.22 (1.10)	< 0.001	-3.31 (1.07)	< 0.001
**Log (BEX), Mean (SD)**		-1.89 (0.70)	-1.64 (0.90)	< 0.001		-1.57 (0.85)	< 0.001	-1.64 (0.91)	< 0.001

**Abbreviations**: BMI, body mass index; BEX: the sum of benzene, ethylbenzene, m-/p-and o-xylene concentrations;

Hearing loss was defined as participants considered to have hearing loss if their hearing thresholds were higher than 25 dB in either ear at any frequency (500, 1000, 2000, 3000, 4000, 6000, and 8000 Hz)

Speech-frequency hearing loss (SFHL) was defined as higher than 25 dB in either ear at any frequency (500, 1,000, 2,000, and 4,000 Hz);

High-frequency hearing loss (HFHL) was defined as higher than 25 dB in either ear at any frequency (3,000, 4,000, 6,000, and 8,000 Hz);

PIR: family income-poverty ratio;

*For continuous variables, *P*-values were calculated using Student’s *t* test, and for categorical variables, *P*-values were computed using chi-square tests

### Associations between BEX and HL

To investigate potential associations between hearing loss and blood BEX levels, we conducted weighted logistic regression analyses (Table 2). In the analysis with hearing loss as the outcome, exposure to ethylbenzene, m-/p-xylene, o-xylene, benzene, and overall BEX increased the risk of occurrence (OR 1.36, 1.22, 1.42, 1.23, and 1.31, respectively; all *P* < 0.05). In the analysis with SFHL as the outcome, ethylbenzene, m-/p-xylene, o-xylene, benzene, and overall BEX increased the risk (OR 1.26, 1.21, 1.28, 1.20, and 1.25, respectively; all *P* < 0.05). For HFHL, exposure to ethylbenzene, m-/p-xylene, o-xylene, benzene, and overall BEX increased the risk (OR 1.36, 1.22, 1.42, 1.22, and 1.31, respectively; all *P* < 0.05).

**Table 2:** Multivariate weighted logistics model analysis reveals the association between the blood log-transformed volume-based-BEX and hearing loss

Characteristics	Hearing loss			SFHL			HFHL	
	Model 1 OR (95%CI)	Model 2 OR (95% CI)		Model 1 OR (95% CI)	Model 2 OR (95% CI)		Model 1 OR (95% CI)	Model 2 OR (95% CI)
Ethylbenzene	**1.49** **[1.25,1.78]**	**1.36** **[1.10,1.68]**		**1.43** **[1.19,1.71]**	**1.26** **[1.04,1.52]**		**1.48** **[1.24,1.75]**	**1.36** **[1.11,1.16]**
M-/p-Xylene	**1.31** **[1.13,1.53]**	**1.22** **[1.02,1.47]**		**1.33** **[1.14,1.55]**	**1.21** **[1.03,1.42]**		**1.30** **[1.12,1.52]**	**1.22** **[1.03,1.46]**
O-Xylene	**1.56** **[1.21,2.01]**	**1.42** **[1.06,1.91]**		**1.43** **[1.16,1.77]**	**1.28** **[1.05,1.56]**		**1.53** **[1.21,1.94]**	**1.42** **[1.07,1.87]**
Benzene	**1.31** **[1.16,1.49]**	**1.23** **[1.05,1.43]**		**1.35** **[1.18,1.54]**	**1.20** **[1.01,1.42]**		**1.31** **[1.15,1.49]**	**1.22** **[1.04,1.44]**
BEX	**1.42** **[1.21,1.67]**	**1.31** **[1.08,1.59]**		**1.41** **[1.20,1.66]**	**1.25** **[1.05,1.49]**		**1.41** **[1.20,1.66]**	**1.31** **[1.09,1.59]**

**Abbreviations**: CI, confidence interval; OR, odds ratio; BEX: the sum of benzene, ethylbenzene, m-/p-and o-xylene concentrations; SFHL: Speech-frequency hearing loss; HFHL: High-frequency hearing loss

Model 1: adjusted for age, sex, race, BMI,

Model 2: adjusted for variables in Model 1 plus diabetes status, drinking status, hypertension, education level, smoking status, marital status, PIR, work noise, recreational noise, firearm noise, and year cycle

Table 3 presents the results of the multivariate weighted ordered logistic regression between BEX and different degrees of hearing loss. The results indicate a positive correlation between the severity of hearing loss and the concentration of BEX and its components. Interestingly, such a relationship was observed only in HL and HFHL; in SFHL, there was no association between the severity of hearing loss and the concentration of BEX and its components. Supplementary Tables 3–4 summarize the results of the second and third sensitivity analyses, revealing that the impact of BEX and its components on hearing loss remains robust.

**Table 3:** Multivariate weighted ordered logistics model used for sensitivity analysis on the association between blood log-BEX and hearing loss

Characteristics	Model 1	Intercepts	
**Hearing loss**	OR (95%CI)	Normal vs. Mild	Mild vs. Moderate to severe
Ethylbenzene	**1.24 [0.04,0.39]**	1.99	4.16
M-/p-Xylene	**1.19 [0.02,0.34]**	2.39	4.56
O-Xylene	**1.27 [0.03,0.44]**	1.98	4.14
Benzene	**1.17 [0.01,0.03]**	2.17	4.34
BEX	**1.24 [0.04,0.39]**	2.43	4.60
**SFHL**			
Ethylbenzene	1.07 [-0.10,0.24]	3.82	5.39
M-/p-Xylene	1.04 [-0.13,0.21]	3.97	5.54
O-Xylene	1.07 [-0.12,0.25]	3.84	5.41
Benzene	1.09 [-0.07,0.22]	3.75	5.32
BEX	1.07 [-0.10,0.23]	3.86	5.53
**HFHL**			
Ethylbenzene	**1.25 [0.05,0.40]**	1.97	4.11
M-/p-Xylene	**1.21 [0.03,0.35]**	2.37	4.52
O-Xylene	**1.28 [0.05,0.45]**	1.95	4.10
Benzene	**1.18 [0.01,0.31]**	2.17	4.32
BEX	**1.25 [0.05,0.40]**	2.42	4.57

**Abbreviations**: CI, confidence interval; OR, odds ratio. SFHL: Speech-frequency hearing loss; HFHL: High-frequency hearing loss

Model 1: adjusted for age, sex, race, BMI, diabetes status, drinking status, hypertension, education level, smoking status, Marital status, PIR, work noise, recreational noise, firearm noise, and year cycle

Log-BEX: log-transformed volatile organic aromatic compound values

Participants were reclassified into three levels based on the average hearing threshold (PTA): normal (< 20 dB), mild (20–35 dB), and moderate to severe (> 35 dB)

Intercepts: represent estimated intercepts for different outcome levels in the ordered logistic regression model

### Subgroup analyses

**Fig. 2:**
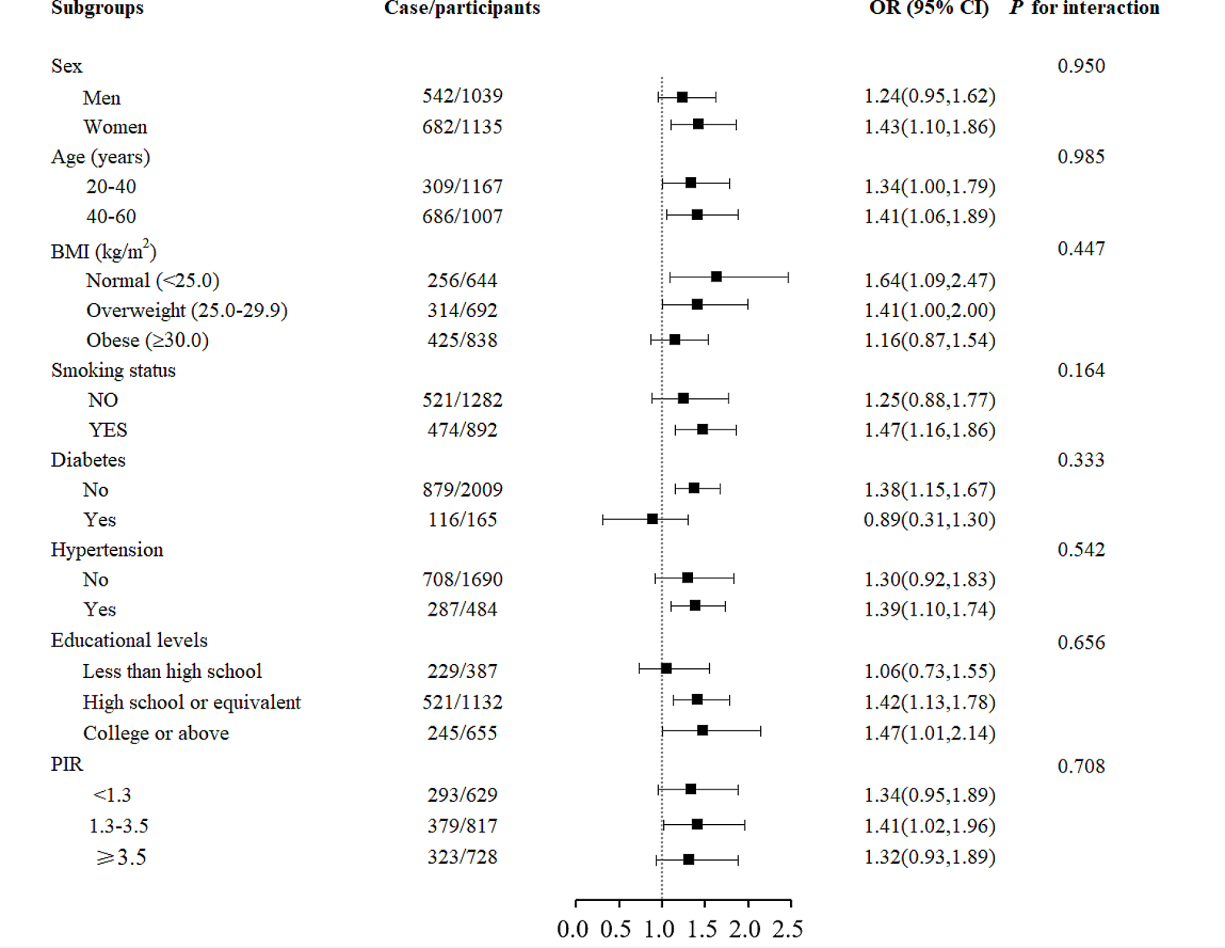
Association between log-transformed creatinine-corrected BEX and hearing loss in subgroups. Models were adjusted for age (20–40 and 40–60 years), sex (male and female), BMI categories (< 25, 25-29.9, and ≥ 30 kg/m^2^), education levels (less than high school, high school, and college or above), race/ethnicity (Mexican American, Other Hispanic, Non-Hispanic White Non-Hispanic Black, Other Race - Including Multi-Racial), marital status (married, widowed, divorced, separated, never married, living with partner), PIR (< 1.3, 1.3–3.5, > 3.5), smoking status (no and yes), drinking status(no and yes), diabetes (no and yes), hypertension (no and yes), work noise (no and yes), recreational noise (no and yes), firearm noise(no and yes), and year cycle (2003–2004, 2011–2012, and 2015–2016). *P* values for interaction were estimated by adding a product term of each stratifying variable and BEX in the main model and assessing it via a Wald test. Abbreviations: BMI, body mass index; CI, confidence interval; OR, odds ratio; PIR, family income-poverty ratio

Figure 2 illustrates a subgroup analysis of BEX based on gender, age, BMI, smoking status, diabetes, hypertension, education level, and PIR. No significant differences were observed among the various components. Additionally, Supplementary Figs. 1–4 present subgroup analysis results for Ethylbenzene, M-/P-Xylene, O-Xylene, and Benzene, respectively. No significant differences were found among the components in these subanalyses. Refer to the supplementary figures for detailed results.

### The cubic spline of the association between log-transformed volume-based BEX and risk of hearing loss

**Fig. 3:**
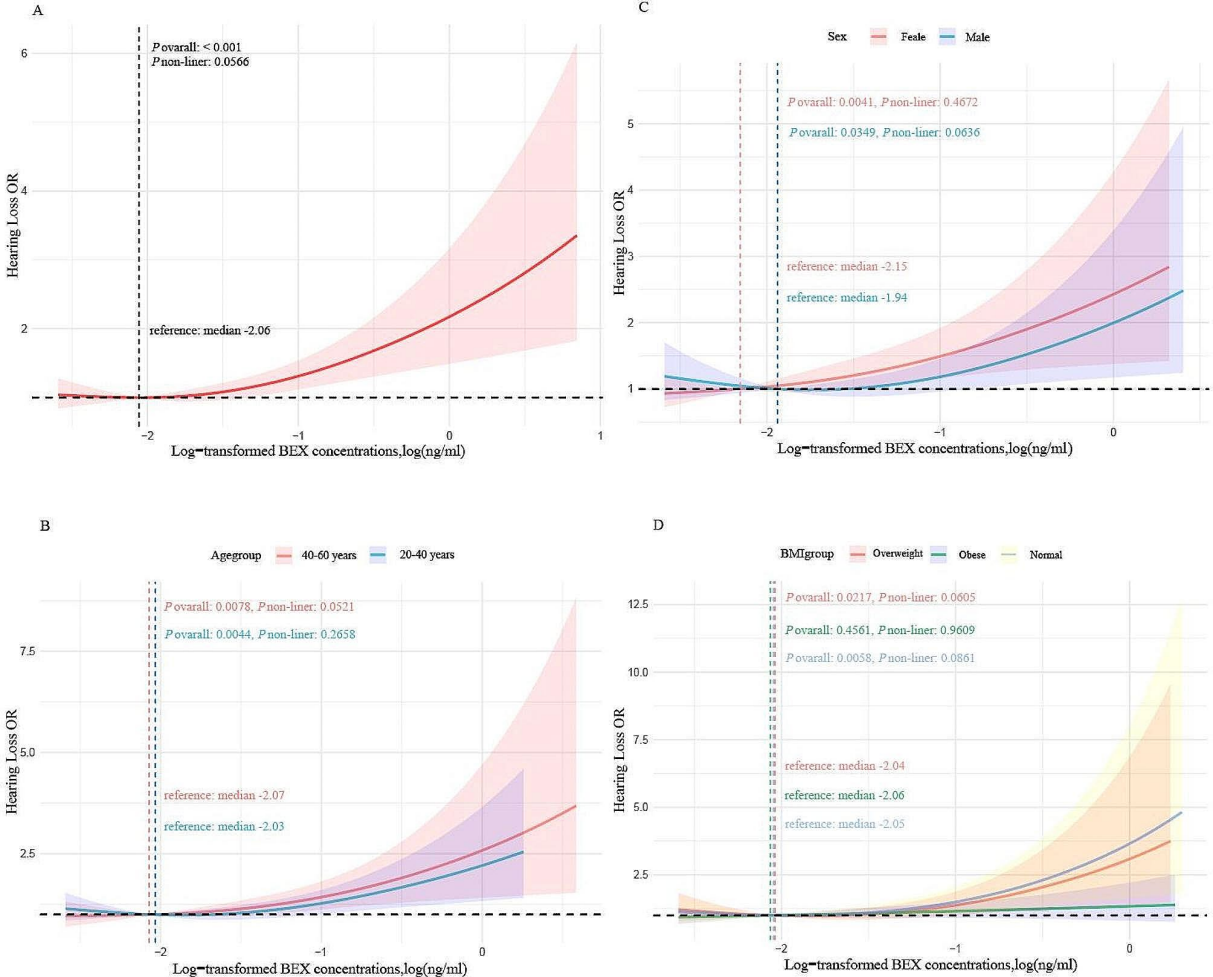
The cubic spline of the association between log-transformed volume-based BEX and risk of hearing loss. (A) BEX and HL; (B) BEX and HL stratified by age; (C) BEX and HL stratified by sex; (D) BEX and HL stratified by BMI. Models were adjusted for age (continuous, years), sex (male and female), BMI (continuous, kg/m2), education levels (less than high school, high school and college or higher), race/ethnicity (Mexican American, Other Hispanic, Non-Hispanic White Non-Hispanic Black, Other Race - Including Multi-Racial), marital status (married, widowed, divorced, separated, never married, living with partner), PIR (< 1.3, 1.3–3.5, > 3.5), smoking status (no and yes), drinking status(no and yes), diabetes (no and yes), hypertension (no and yes), work noise(no and yes), recreational noise(no and yes), firearm noise(no and yes), and year cycle (2003–2004, 2011–2012, and 2015–2016). Knots = 3. Abbreviations: BMI, body mass index; PIR, family income-poverty ratio

In accordance with the fully adjusted model, we employed a constrained cubic spline method to investigate the dose-response relationship between the concentrations of BEX and its components and the incidence of hearing loss (refer to Fig. 3). The outcomes illuminate a linear correlation between the logarithmic concentration of BEX and the susceptibility to hearing loss (*P*-linear-value < 0.05). Furthermore, upon stratification by distinct age groups, genders, and BMI categories, the dose-response relationship suggests a comparatively higher risk among individuals of older age, female gender, and those with normal weight. Notably, no evidence of a nonlinear correlation was observed between the logarithmically transformed concentrations of ethylbenzene, ortho-xylene, meta/para-xylene, benzene, and hearing loss (*P*-linear-value < 0.05; Supplementary Figs. 5–8).

## Discussion

In this study targeting US adults, we observed a positive correlation between concentrations of volatile organic compounds, including benzene, ethylbenzene, o-xylene, and m/p-xylene, in blood samples from diverse populations and various types of hearing loss in American adults, including overall hearing loss, low-frequency hearing loss, and high-frequency hearing loss.

Currently, effective treatments for hearing loss remain limited, emphasizing the significance of primary prevention to mitigate and reduce risk factors. Multiple studies, conducted through both animal experimentation and epidemiological research, have explored the potential correlation between BEX exposure and hearing loss. In rat animal models, exposure to ethylbenzene induces apoptosis in cochlear precursor cells, inhibits cell proliferation, and alters mitochondrial membrane potential, affecting hearing. Additionally, combined exposure to toluene and ethylbenzene leads to enhanced outer hair cell death, resulting in auditory function loss [45, 46]. Previous investigations have explored the occupational risks of toluene, ethylbenzene, xylene, and styrene (TEXS) exposure concerning hearing loss among petrochemical workers [47]. Other studies have found an association between organic solvents (benzene, toluene, and xylene) and the incidence of high-frequency hearing loss [48]. However, each of these studies has certain limitations. Some are confined to occupational cohorts, others focus solely on individual component impacts, while some solely concentrate on the overall effects of TEXS. This study further reveals that exposure to BEX in the bloodstream is associated with an elevated risk of hearing loss, impacting both speech frequency and high-frequency auditory function.

The organ of the Corti, situated within the cochlea, serves as the central hub for encoding electrical signals in the auditory system. The Organ of Corti’s is quite a complex one. During sound-induced hearing, vibrations from the stapes are propagated by the traveling wave phenomenon to the different spectral components’ tonotopic locations along the basilar membrane (BM). The traveling wave derives from the interaction between the basilar membrane elastic structure and the fluid differential pressure developed between the scala vestibuli and the scala Tympani. The true mechano-electrical transduction happens at the level of the inner hair cells (IHC), in which the signal is conveyed to the spiral ganglion. The outer hair cells (OHC) are responsible for the amplification of the mechanical vibration of the basilar membrane at low stimulus levels. The somato-elasticity of the OHC provides an electromechanical stage of amplification. The transmembrane potential of the OHC is triggered by the hair bundle displacement against the tectorial membrane. This mechanism provides a mechano-electrical piezoelectric stage. Due to this complexity in the sound transduction the interplay among hair cells, both inner hair cells and outer hair cells, vascular structures, and the spiral ganglion neurons is indispensable to formulate hypotheses about the BEX–cochlea interaction in inducing hearing loss. Notably, exposure to ethylbenzene in mice was observed to hinder the Wnt/β-catenin signaling pathway, leading to mitochondrial abnormalities in cochlear progenitor cells, resulting in excessive apoptosis and contributing indirectly to hearing loss [49]. Moreover, ethylbenzene exposure has been linked to altered neurotransmitter profiles and hearing loss [50]. The α9/α10 nicotinic acetylcholine receptors, predominantly expressed in the mammalian cochlea, mediate synaptic transmission between hair cells and spiral ganglion neurons [51]. At low concentrations, both ethylbenzene and m-xylene (10 µM) inhibit the opening of α9/α10 nAChR ion channels, affecting synaptic signal transmission and resulting in auditory impairment [52]. A study exploring the toxicity of aromatic solvents on the organ of Corti revealed that ethylbenzene exhibited more pronounced cytotoxicity than o-xylene, inducing damage to both outer hair cells and first-row inner hair cells [53]. 

Not all components of BEX have been thoroughly studied for their impact on hearing loss. Studies investigating the effects of benzene and the three isomers of xylene on hearing loss in rats have found that only m-/p-xylene has ototoxic effects on hair cells [54, 55]. However, our research indicates that both benzene and ortho/meta-xylene have an impact on human hearing loss. Literature review reveals that ortho/meta-xylene can induce ear edema, oxidative stress, and inflammation, while also causing constriction or damage to vascular endothelium, and para-xylene can reduce potassium ion concentration in lymph fluid [56, 57]. These factors could potentially hinder blood supply to the cochlea or lymphatic reflux, ultimately leading to dysfunction of the cochlear organ. Although there is no explicit experimental evidence of the ototoxic effects of benzene, prolonged exposure to low doses of benzene can activate cellular oxidative stress, which may contribute to hearing loss [58]. Furthermore, the animal models used for studying ortho/meta-xylene and benzene may have species-specific variations. Further research is necessary to elucidate the specific mechanisms underlying hearing loss associated with the isomers of benzene and xylene.

It is noteworthy that the restricted cubic spline plots reveal a higher risk in the 40–60 age group compared to the 20–40 age group. This contradicts the results of subgroup analysis, and some factors contributing to this discrepancy can be partly explained by the design of our study and the relatively small sample size. Age and other demographic factors (gender, race/ethnicity, educational level) are important influencing factors contributing to hearing impairment [59]. Therefore, potential factor could be the ongoing increase in the contribution of exposure to other environmental risk factors, such as cadmium, lead, and other heavy metals, which may continuously augment the impact of BEX that we have been investigating on hearing loss [10]. Furthermore, there exists the possibility of heterogeneity introduced by our definition of hearing loss (> 25dB) in the 20–60 age group. However, this definition of hearing loss (> 25dB) still applies in numerous age-related studies of hearing loss, particularly in the speech frequency range [59–61]. To better address this heterogeneity, we conducted sensitivity analyses. Our results remained robust even after excluding individuals over the age of 40, redefining hearing loss, and controlling for serum exposure to cotinine. In conclusion, future studies necessitate more samples, longer follow-up periods, and foundational experiments to explore the relationship between age, BEX, and hearing loss.

Additionally, individuals with obesity exhibit a lower risk from BEX compared to those with normal weight, while females are more susceptible than males. These two phenomena might be attributed to Insulin-like Growth Factor 1 (IGF-1). Studies suggest that IGF-1, acting as a neurotrophic factor, can enhance the antioxidant response of cochlear hair cells via the IGF1R/AKT pathway, thus protecting auditory cells from oxidative stress and cell apoptosis [62]. For obese patients, the secretion of IGF-1 is complex and subject to controversy. Some studies suggest that free IGF-1 increases during obesity and promotes the expression of IGFR (Insulin-like Growth Factor Receptor). However, obese individuals with metabolic disturbances and associated complications tend to have lower levels of free IGF-1 than the absolute levels would suggest [63, 64]. This phenomenon might be related to the RCS (restricted cubic spline) curve we derived, indicating that higher levels of IGF-1 and IGFR could result in a lower risk of hearing loss induced by BEX in obese individuals compared to normal-weight individuals. However, overweight patients might experience a partial failure of protective factors due to a more disrupted endocrine system, leading to a risk lying between that of normal-weight individuals and obese patients. This speculation is based on our analysis of the research results. As it is a cross-sectional study, further experiments may be necessary to ascertain whether obesity can indeed prevent hearing loss. Abdel Halim Harrath and colleagues’ research indicates that exposure to benzene and ethylbenzene can significantly decrease the levels of IGF-1 in female rats, while also increasing the secretion of estrogen and progesterone [13]. Therefore, the difference in risk between men and women may not be limited solely to IGF-1, but also associated with sex hormones. Estrogen and progesterone are primarily secreted by the ovaries in female individuals, and in addition to their role in reproductive system maintenance, they also exert protective effects on the auditory system. Estrogen receptors are widely distributed in the cochlea and vascular endothelium, promoting vascular growth and safeguarding against hearing loss [65, 66]. Nevertheless, studies by Kassotis and colleagues have reported antagonistic activity of BEX on estrogen receptors [67]. Prolonged combined stimulation of estrogen and progesterone in females can elevate hearing thresholds, leading to negative impacts on hearing [68]. Additionally, expression of aquaporin 5, a water channel protein in the cochlea, can be influenced by uterine estrogen, promoting the movement of cellular fluid to interstitial tissue, thus causing water and sodium retention effects that could result in cochlear vascular microcirculation or lymphatic congestion, ultimately affecting hearing [69]. Therefore, we hypothesize that BEX may contribute to the disruption of the female endocrine system. Although compensatory increases in estrogen may occur, the negative effects resulting from estrogen imbalance far outweigh its protective benefits.

Our study possesses several strengths: benzene, ethylbenzene, m-/p-xylene, and o-xylene, as major components of volatile organic compounds in human blood, are positively associated with hearing loss in the adult population of the United States. Consistent conclusions were also observed for both low-frequency and high-frequency hearing loss. Future research should delve deeper into the mechanisms of organic compounds, especially BEX, in causing hearing loss, to validate these findings longitudinally within the environmental context. Nevertheless, our study has inherent limitations. First, its cross-sectional nature restricts the ability to infer causality between exposure and outcomes. Additionally, the measurement of volatile organic compounds in the blood is temporary and may not represent long-term BEX exposure levels. Lastly, our study focuses on BEX exposure and does not account for the potential effects of other hazardous substances.

## Conclusions

Our study indicated a positive correlation between individual or cumulative exposure to benzene, ethylbenzene, meta/para-xylene, and ortho-xylene and the risk of HL, SFHL, and HFHL. Further research is imperative to acquire a more comprehensive understanding of the mechanisms by which organic compounds, notably BEX, cause hearing loss and to validate these findings in longitudinal environmental studies.

### Electronic supplementary material

Below is the link to the electronic supplementary material.

Supplementary Material 1

Supplementary Material 2

## Data Availability

Data described in the manuscript are publicly and freely available without restriction at: https://www.cdc.gov/nchs/nhanes/index.htm.
