# Supplementary material for: Association between blood volatile organic aromatic compound concentrations and hearing loss in US adults
Source: BMC Public Health. 2024 Feb 27;24:623. doi: 10.1186/s12889-024-18065-0 (PMC10897984; doi:10.1186/s12889-024-18065-0)
Supplement: Supplementary file 1 — Supplementary Material 1 [file 12889_2024_18065_MOESM1_ESM.docx]

**Supplementary Table 1. Baseline characteristics of study participants in NHANES 2003–2004,2011-2012 and 2015-2016 according to SFHL.**

| **Characteristics** | **SFHL** | |  |
| --- | --- | --- | --- |
|  | **NO (N = 1661)** | **YES (N = 513)** | ****P*-value** |
| **Age, Mean (SD)** | 36.63 (10.86) | 46.83 (10.00) | <0.001 |
| **BMI, Mean (SD)** | 28.75 (7.09) | 29.80 (6.05) | 0.036 |
| **Gender, n, %** |  |  | <0.001 |
| Male | 692 (41.9%) | 347 (67.1%) |  |
| Female | 969 (58.1%) | 166 (32.9%) |  |
| **Race, n, %** |  |  | 0.012 |
| Mexican American | 239 (8.6%) | 80 (8.0%) |  |
| Other Hispanic | 165 (6.9%) | 52 (5.1%) |  |
| Non-Hispanic White | 648 (65.6%) | 233 (73.3%) |  |
| Non-Hispanic Black | 343 (11.1%) | 73 (6.2%) |  |
| Other Race - Including Multi-Racial | 266 (7.8%) | 75 (7.4%) |  |
| **Educational levels, n, %** |  |  | <0.001 |
| Less than high school | 257 (10.4%) | 130 (16.6%) |  |
| High school or equivalent | 854 (50.3%) | 278 (57.1%) |  |
| College or above | 550 (39.3%) | 105 (26.3%) |  |
| **Marital status, n, %** |  |  | <0.001 |
| Married | 809 (51.0%) | 298 (59.9%) |  |
| Widowed | 15 (0.7%) | 9 (1.6%) |  |
| Divorced | 113 (6.6%) | 60 (12.5%) |  |
| Separated | 55 (2.3%) | 22 (3.2%) |  |
| Never married | 464 (27.5%) | 73 (14.4%) |  |
| Living with partner | 205 (11.9%) | 51 (8.4%) |  |
| **Smoking status, n, %** |  |  | <0.001 |
| NO | 1053 (61.5%) | 229 (44.5%) |  |
| YES | 608 (38.5%) | 284 (55.5%) |  |
| **Drinking status, n, %** |  |  | 0.018 |
| NO | 1178 (76.7%) | 334 (69.8%) |  |
| YES | 483 (23.3%) | 179 (30.2%) |  |
| **Hypertension, n, %** |  |  | <0.001 |
| NO | 1342 (82.9%) | 348 (68.6%) |  |
| YES | 319 (17.1%) | 165 (31.4%) |  |
| **Diabetes, n, %** |  |  | <0.001 |
| NO | 1063 (95.5%) | 446 (89.3%) |  |
| YES | 98 (4.5%) | 67 (10.7%) |  |
| **PIR, n, %** |  |  | 0.768 |
| <1.3 | 471 (21.2%) | 158 (20.0%) |  |
| 1.3-3.5 | 618 (35.1%) | 199 (37.0%) |  |
| ≥3.5 | 572 (43.7%) | 156 (43.0%) |  |
| **Noise exposure, yes, n, %** |  |  |  |
| Work noise | 208 (12.9%) | 110 (21.9%) | <0.001 |
| Recreational noise | 252 (15.7%) | 109 (22.2%) | 0.012 |
| Firearm noise | 540 (40.9%) | 205 (47.5%) | 0.014 |
| **Year cycle, n, %** |  |  | 0.678 |
| 2003-2004 | 303 (19.5%) | 99 (21.5%) |  |
| 2011-2012 | 558 (34.7%) | 166 (32.6%) |  |
| 2015-2016 | 800 (45.8%) | 248 (45.9%) |  |
| **Log (ethylbenzene), Mean (SD)** | -3.68 (0.70) | -3.43 (0.77) | <0.001 |
| **Log (m-/p-Xylene), Mean (SD)** | -2.67 (0.90) | -2.37 (0.93) | <0.001 |
| **Log(o-Xylene), Mean (SD)** | -3.69 (0.61) | -3.51 (0.64) | <0.001 |
| **Log (benzene), Mean (SD)** | -3.52 (0.89) | -3.22 (1.10) | <0.001 |
| **Log (BEX), Mean (SD)** | -1.84 (0.78) | -1.57 (0.85) | <0.001 |

**Abbreviations:** BMI, body mass index; BEX: the sum of benzene, ethylbenzene, m-/p-and o-xylene concentrations;

Speech-frequency hearing loss (SFHL) was defined as higher than 25 dB in either ear at any frequency (500, 1,000, 2,000, and 4,000 Hz).

PIR: family income-poverty ratio;

*For continuous variables, *P*-values were calculated using Student's *t* test, and for categorical variables, *P*-values were computed using chi-square tes

**Supplementary Table 2. Baseline characteristics of study participants in NHANES 2003–2004,2011-2012 and 2015-2016 according to HFHL.**

| **Characteristics** | **HFHL** | |  |
| --- | --- | --- | --- |
|  | **NO (N = 1201)** | **YES (N = 973)** | ****P*-value** |
| **Age, Mean (SD)** | 34.43 (9.99) | 44.83 (10.66) | <0.001 |
| **BMI, Mean (SD)** | 28.43 (7.12) | 29.69 (6.48) | 0.004 |
| **Gender, n, %** |  |  | <0.001 |
| Male | 503 (42.5%) | 536 (55.1%) |  |
| Female | 698 (57.5%) | 437 (44.9%) |  |
| **Race, n, %** |  |  | 0.117 |
| Mexican American | 173 (8.9%) | 146 (8.0%) |  |
| Other Hispanic | 110 (6.6%) | 107 (6.2%) |  |
| Non-Hispanic White | 477 (65.4%) | 404 (70.0%) |  |
| Non-Hispanic Black | 252 (11.4%) | 164 (8.1%) |  |
| Other Race - Including Multi-Racial | 189 (7.7%) | 152 (7.7%) |  |
| **Educational levels, n, %** |  |  | <0.001 |
| Less than high school | 161 (9.1%) | 226 (15.3%) |  |
| High school or equivalent | 624 (50.8%) | 508 (53.5%) |  |
| College or above | 416 (40.1%) | 239 (31.2%) |  |
| **Marital status, n, %** |  |  | <0.001 |
| Married | 548 (47.6%) | 559 (59.8%) |  |
| Widowed | 5 (0.6%) | 19 (1.4%) |  |
| Divorced | 64 (4.7%) | 109 (12.0%) |  |
| Separated | 39 (2.3%) | 38 (2.8%) |  |
| Never married | 378 (31.2%) | 159 (16.1%) |  |
| Living with partner | 167 (13.6%) | 89 (7.9%) |  |
| **Smoking status, n, %** |  |  | <0.001 |
| NO | 776 (62.7%) | 506 (50.6%) |  |
| YES | 425 (37.3%) | 467 (49.4%) |  |
| **Drinking status, n, %** |  |  | 0.055 |
| NO | 872 (77.2%) | 640 (72.3%) |  |
| YES | 329 (22.8%) | 333 (27.7%) |  |
| **Hypertension, n, %** |  |  | <0.001 |
| NO | 1001 (84.8%) | 689 (72.7%) |  |
| YES | 200 (15.2%) | 284 (27.3%) |  |
| **Diabetes, n, %** |  |  | <0.001 |
| NO | 1152 (96.4%) | 857 (81.1%) |  |
| YES | 49 (3.6%) | 116 (8.9%) |  |
| **PIR, n, %** |  |  | 0.604 |
| <1.3 | 342 (21.8%) | 287 (18.9%) |  |
| 1.3-3.5 | 450 (35.6%) | 367 (35.6%) |  |
| ≥3.5 | 409 (42.6%) | 319 (45.5 %) |  |
| **Noise exposure, yes, n, %** |  |  |  |
| Work noise | 145 (12.5%) | 338 (18.3%) | 0.759 |
| Recreational noise | 186 (15.8%) | 175 (19.1%) | 0.161 |
| Firearm noise | 407 (42.2%) | 173 (43.0%) | 0.015 |
| **Year cycle, n, %** |  |  | 0.931 |
| 2003-2004 | 232 (20.2%) | 170 (19.7%) |  |
| 2011-2012 | 394 (33.6%) | 330 (34.8%) |  |
| 2015-2016 | 575 (46.2%) | 473 (45.5%) |  |
| **Log (ethylbenzene), Mean (SD)** | -3.72 (0.61) | -3.49 (0.83) | <0.001 |
| **Log (m-/p-Xylene), Mean (SD)** | -2.71 (0.83) | -2.46 (0.99) | <0.001 |
| **Log(o-Xylene), Mean (SD)** | -3.73 (0.53) | -3.55 (0.70) | <0.001 |
| **Log (benzene), Mean (SD)** | -3.56 (0.82) | -3.31 (1.07) | <0.001 |
| **Log (BEX), Mean (SD)** | -1.89 (0.70) | -1.64 (0.91) | <0.001 |

**Abbreviations:** BMI, body mass index; BEX: the sum of benzene, ethylbenzene, m-/p-and o-xylene concentrations;

High-frequency hearing loss (HFHL) was defined as higher than 25 dB in either ear at any frequency (3,000, 4,000, 6,000, and 8,000 Hz);

PIR: family income-poverty ratio;

*For continuous variables, *P*-values were calculated using Student's t test, and for categorical variables, *P*-values were computed using chi-square test

**Supplementary Table 3. Multivariate weighted logistics model used for sensitivity analysis on the association between blood log-BEX and hearing loss (Exclude participants aged over 40 years).**

| Characteristics | Hearing loss |  |  | SFHL |  |  | HFHL |  |
| --- | --- | --- | --- | --- | --- | --- | --- | --- |
|  | Model 1  OR (95%CI) | Model 2  OR (95% CI) |  | Model 1  OR (95% CI) | Model 2  OR (95% CI) |  | Model 1  OR (95% CI) | Model 2  OR (95% CI) |
| Ethylbenzene | **1.55**  **[1.27,1.89]** | **1.37**  **[1.09,1.72]** |  | **1.46**  **[1.07,2.00]** | **1.36**  **[1.04,1.78]** |  | **1.52**  **[1.25,1.84]** | **1.35**  **[1.08,1.70]** |
| M-/p-Xylene | **1.37**  **[1.14,1.64]** | 1.26  [0.99,1.59] |  | **1.39**  **[1.07,1.80]** | 1.27  [0.99,1.63] |  | **1.35**  **[1.12,1.61]** | 1.25  [0.99,1.58] |
| O-Xylene | **1.60**  **[1.20,2.13]** | **1.42**  **[1.01,2.00]** |  | **1.49**  **[1.07,2.08]** | **1.37**  **[1.02,1.85]** |  | **1.58**  **[1.19,2.09]** | **1.43**  **[1.02,2.01]** |
| Benzene | **1.44**  **[1.20,1.72]** | **1.36**  **[1.09,1.68]** |  | **1.43**  **[1.12,1.83]** | **1.37**  **[1.02,1.82]** |  | **1.41**  **[1.18,1.68]** | **1.33**  **[1.07,1.64]** |
| BEX | **1.52**  **[1.24,1.86]** | **1.38**  **[1.07,1.77]** |  | **1.48**  **[1.12,1.97]** | **1.36**  **[1.04,1.77]** |  | **1.49**  **[1.22,1.82]** | **1.36**  **[1.06,1.75]** |

**Abbreviations:** CI, confidence interval; OR, odds ratio; BEX: the sum of benzene, ethylbenzene, m-/p-and o-xylene concentrations; SFHL: Speech-frequency hearing loss; HFHL: High-frequency hearing loss.

Model 1: adjusted for age, sex, race, BMI,

Model 2: adjusted for variables in Model 1 plus diabetes status, drinking status, hypertension, education level, smoking status, marital status, PIR, work noise, recreational noise, firearm noise, and year cycle.

**Supplementary Table 4. Multivariate weighted logistics model used for sensitivity analysis on the association between blood log-BEX and hearing loss (Controlling for whether the individual is an acute ETS (Environmental Tobacco Smoke) exposure subject).**

| Characteristics | Hearing loss | | SFHL | |  | | HFHL | | |
| --- | --- | --- | --- | --- | --- | --- | --- | --- | --- |
|  | Model 2  OR (95% CI) |  | | Model 2  OR (95% CI) | |  | | Model 2  OR (95% CI) |  |
| Ethylbenzene | **1.36**  **[1.10,1.68]** |  | | **1.24**  **[1.03,1.50]** | |  | | **1.36**  **[1.11,1.67]** |  |
| M-/p-Xylene | **1.22**  **[1.02,1.46]** |  | | **1.19**  **[1.01,1.41]** | |  | | **1.23**  **[1.04,1.46]** |  |
| O-Xylene | **1.44**  **[1.07,1.94]** |  | | **1.26**  **[1.04,1.54]** | |  | | **1.44**  **[1.09,1.91]** |  |
| Benzene | **1.22**  **[1.05,1.43]** |  | | 1.18  [0.99,1.41] | |  | | **1.23**  **[1.04,1.45]** |  |
| BEX | **1.32**  **[1.09,1.59]** |  | | **1.23**  **[1.04,1.47]** | |  | | **1.32**  **[1.10,1.59]** |  |

**Abbreviations:** CI, confidence interval; OR, odds ratio; BEX: the sum of benzene, ethylbenzene, m-/p-and o-xylene concentrations; SFHL: Speech-frequency hearing loss; HFHL: High-frequency hearing loss.

Model 2: adjusted for age, sex, race, BMI, diabetes status, drinking status, hypertension, education level, smoking status, marital status, PIR, work noise, recreational noise, firearm noise, year cycle, and ETS.
