# Supplementary material for: Association between blood volatile organic aromatic compound concentrations and hearing loss in US adults
Source: BMC Public Health. 2024 Feb 27;24:623. doi: 10.1186/s12889-024-18065-0 (PMC10897984; doi:10.1186/s12889-024-18065-0)
Supplement: Supplementary file 2 — Supplementary Material 2 [file 12889_2024_18065_MOESM2_ESM.pdf]

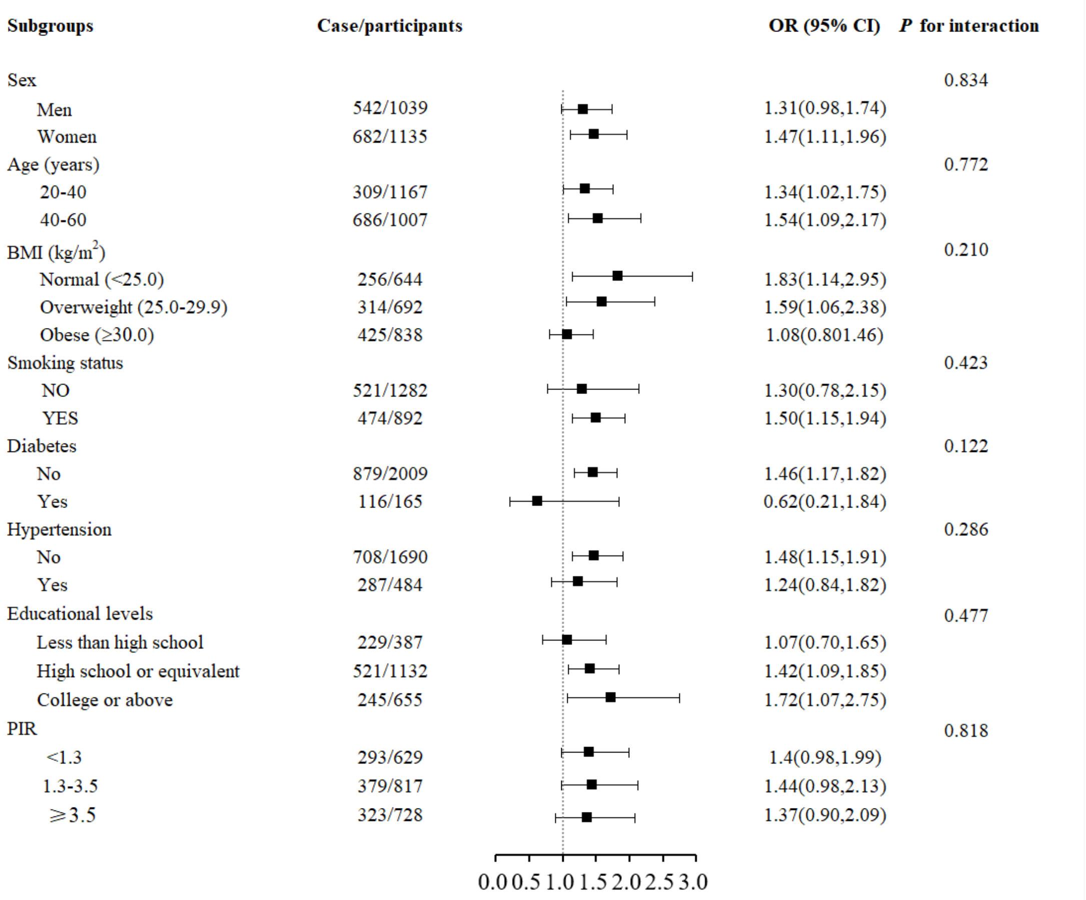

Supplementary Figure 1. Association between log-transformed volume-based Ethylbenzene and risk of hearing loss in subgroups. Models were adjusted for age (20-40 and 40-60 years), sex (male and female), BMI categories (<25, 25-29.9, and ≥30 kg/m^2), education levels (less than high school, high school, and college or above), race/ethnicity (Mexican American, Other Hispanic, Non-Hispanic White Non-Hispanic Black, Other Race - Including Multi-Racial), marital status (married, widowed, divorced, separated, never married, living with partner), PIR (<1.3, 1.3-3.5, >3.5), smoking status (no and yes), drinking status(no and yes), diabetes (no and yes), hypertension (no and yes), work noise (no and yes), recreational noise (no and yes), firearm noise(no and yes), and year cycle (2003-2004, 2011-2012, and 2015-2016). *P* values for interaction were estimated by adding a product term of each stratifying variable and Ethylbenzene in the main model and assessing it via a Wald test. Abbreviations: BMI, body mass index; CI, confidence interval; OR, odds ratio; PIR, family income-poverty ratio.

Subgroups	Case/participants	OR (95% CI)	*P* for interaction
Sex			0.971
Men	542/1039	1.16(0.91,1.47)	
Women	682/1135	1.33(1.06,1.67)	
Age (years)			0.856
20-40	309/1167	1.23(0.95,1.6)	
40-60	686/1007	1.30(1.00,1.69)	
BMI (kg/m^2)			0.493
Normal (<25.0)	256/644	1.49(1.04,2.14)	
Overweight (25.0-29.9)	314/692	1.29(0.95,1.76)	
Obese (≥30.0)	425/838	1.10(0.85,1.44)	
Smoking status			0.071
NO	521/1282	1.17(0.91,1.51)	
YES	474/892	1.37(1.11,1.69)	
Diabetes			0.378
No	879/2009	1.27(1.07,1.51)	
Yes	116/165	0.87(0.37,2.19)	
Hypertension			0.658
No	708/1690	1.27(1.03,1.56)	
Yes	287/484	1.25(0.92,1.70)	
Educational levels			0.795
Less than high school	229/387	1.05(0.76,1.46)	
High school or equivalent	521/1132	1.30(1.05,1.62)	
College or above	245/655	1.30(0.95,1.79)	
PIR			0.389
<1.3	293/629	1.29(0.96,1.73)	
1.3-3.5	379/817	1.31(0.99,1.74)	
≥3.5	323/728	1.20(0.90,1.58)	

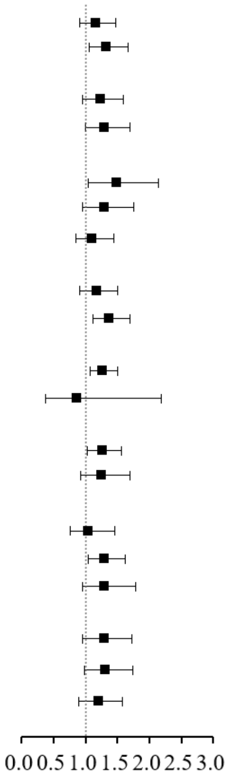

Supplementary Figure 2. Association between log-transformed volume-based M-/P-Xylene and risk of hearing loss in subgroups. Models were adjusted for age (20-40 and 40-60 years), sex (male and female), BMI categories (<25, 25-29.9, and ≥30 kg/m^2), education levels (less than high school, high school, and college or above), race/ethnicity (Mexican American, Other Hispanic, Non-Hispanic White Non-Hispanic Black, Other Race - Including Multi-Racial), marital status (married, widowed, divorced, separated, never married, living with partner), PIR (<1.3, 1.3-3.5, >3.5), smoking status (no and yes), drinking status(no and yes), diabetes (no and yes), hypertension (no and yes), work noise (no and yes), recreational noise (no and yes), firearm noise(no and yes), and year cycle (2003-2004, 2011-2012, and 2015-2016). *P* values for interaction were estimated by adding a product term of each stratifying variable and M-/P-Xylene in the main model and assessing it via a Wald test. Abbreviations: BMI, body mass index; CI, confidence interval; OR, odds ratio; PIR, family income-poverty ratio.

Subgroups	Case/participants	OR (95% CI)	*P* for interaction
Sex			0.894
Men	542/1039	1.29(0.93,1.81)	
Women	682/1135	1.65(1.14,2.38)	
Age (years)			0.809
20-40	309/1167	1.45(0.98,2.13)	
40-60	686/1007	1.60(1.04,2.45)	
BMI (kg/m^2)			0.357
Normal (<25.0)	256/644	2.17(1.23,3.81)	
Overweight (25.0-29.9)	314/692	1.69(1.02,2.82)	
Obese (≥30.0)	425/838	1.11(0.76,1.63)	
Smoking status			0.349
NO	521/1282	1.43(0.87,2.36)	
YES	474/892	1.55(1.13,2.14)	
Diabetes			0.404
No	879/2009	1.52(1.14,2.02)	
Yes	116/165	0.72(0.14,3.67)	
Hypertension			0.292
No	708/1690	1.58(1.12,2.22)	
Yes	287/484	1.22(0.77,1.93)	
Educational levels			0.922
Less than high school	229/387	1.27(0.74,2.18)	
High school or equivalent	521/1132	1.45(1.04,2.04)	
College or above	245/655	1.81(0.98,3.36)	
PIR			0.475
<1.3	293/629	1.63(1.09,2.46)	
1.3-3.5	379/817	1.58(0.95,2.60)	
≥3.5	323/728	1.38(0.86,2.19)	

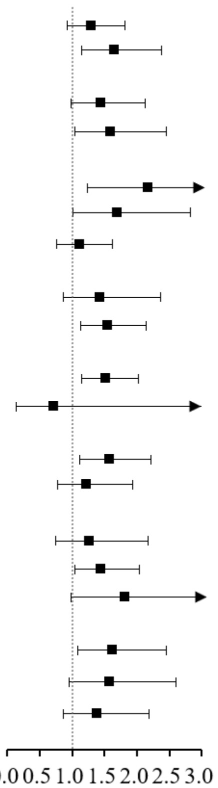

Supplementary Figure 3. Association between log-transformed volume-based O-Xylene and risk of hearing loss in subgroups. Models were adjusted for age (20-40 and 40-60 years), sex (male and female), BMI categories (<25, 25-29.9, and ≥30 kg/m^2), education levels (less than high school, high school, and college or above), race/ethnicity (Mexican American, Other Hispanic, Non-Hispanic White Non-Hispanic Black, Other Race - Including Multi-Racial), marital status (married, widowed, divorced, separated, never married, living with partner), PIR (<1.3, 1.3-3.5, >3.5), smoking status (no and yes), drinking status(no and yes), diabetes (no and yes), hypertension (no and yes), work noise (no and yes), recreational noise (no and yes), firearm noise(no and yes), and year cycle (2003-2004, 2011-2012, and 2015-2016). *P* values for interaction were estimated by adding a product term of each stratifying variable and O-Xylene in the main model and assessing it via a Wald test. Abbreviations: BMI, body mass index; CI, confidence interval; OR, odds ratio; PIR, family income-poverty ratio.

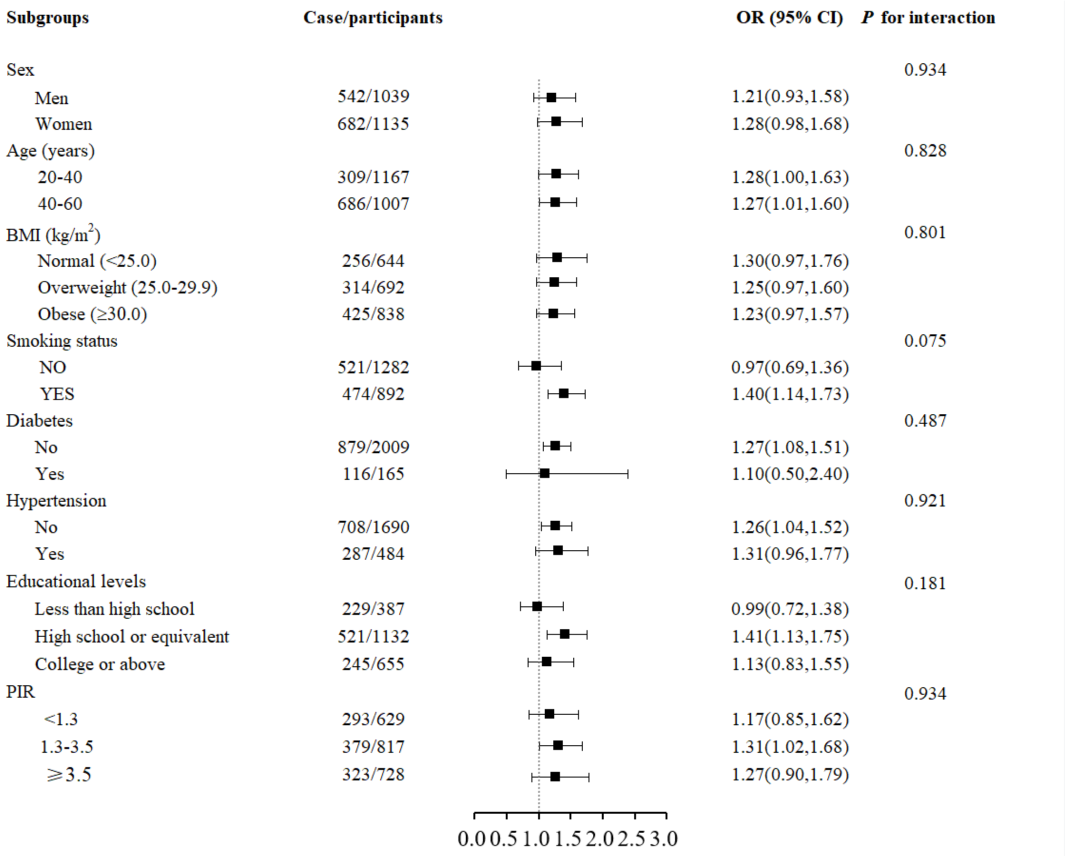

Supplementary Figure 4. Association between log-transformed volume-based Benzene and risk of hearing loss in subgroups. Models were adjusted for age (20-40 and 40-60 years), sex (male and female), BMI categories (<25, 25-29.9, and ≥30 kg/m^2), education levels (less than high school, high school, and college or above), race/ethnicity (Mexican American, Other Hispanic, Non-Hispanic White Non-Hispanic Black, Other Race - Including Multi-Racial), marital status (married, widowed, divorced, separated, never married, living with partner), PIR (<1.3, 1.3-3.5, >3.5), smoking status (no and yes), drinking status(no and yes), diabetes (no and yes), hypertension (no and yes), work noise (no and yes), recreational noise (no and yes), firearm noise(no and yes), and year cycle (2003-2004, 2011-2012, and 2015-2016). *P* values for interaction were estimated by adding a product term of each stratifying variable and Benzene in the main model and assessing it via a Wald test. Abbreviations: BMI, body mass index; CI, confidence interval; OR, odds ratio; PIR, family income-poverty ratio.

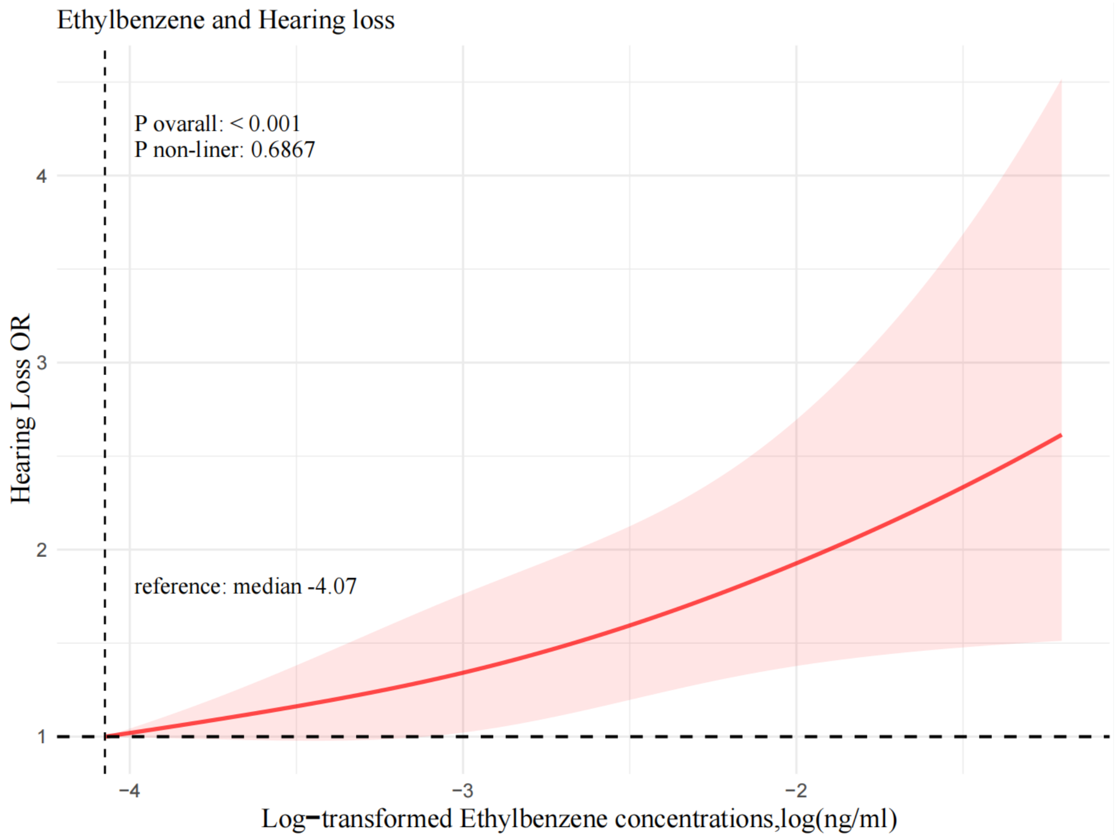

Supplementary Figure 5. The cubic spline of the association between log-transformed volume-based ethylbenzene and risk of hearing loss. Models were adjusted for age (continuous, years), sex (male and female), BMI (continuous, kg/m^2), education levels (less than high school, high school and college or higher), race/ethnicity (Mexican American, Other Hispanic, Non-Hispanic White Non-Hispanic Black, Other Race - Including Multi-Racial), marital status (married, widowed, divorced, separated, never married, living with partner), PIR (<1.3, 1.3-3.5, >3.5), smoking status (no and yes), drinking status(no and yes), diabetes (no and yes), hypertension (no and yes), work noise(no and yes), recreational noise(no and yes), firearm noise(no and yes), and year cycle (2003-2004, 2011-2012, and 2015-2016). Knots =3. Abbreviations: BMI, body mass index; PIR, family income-poverty ratio.

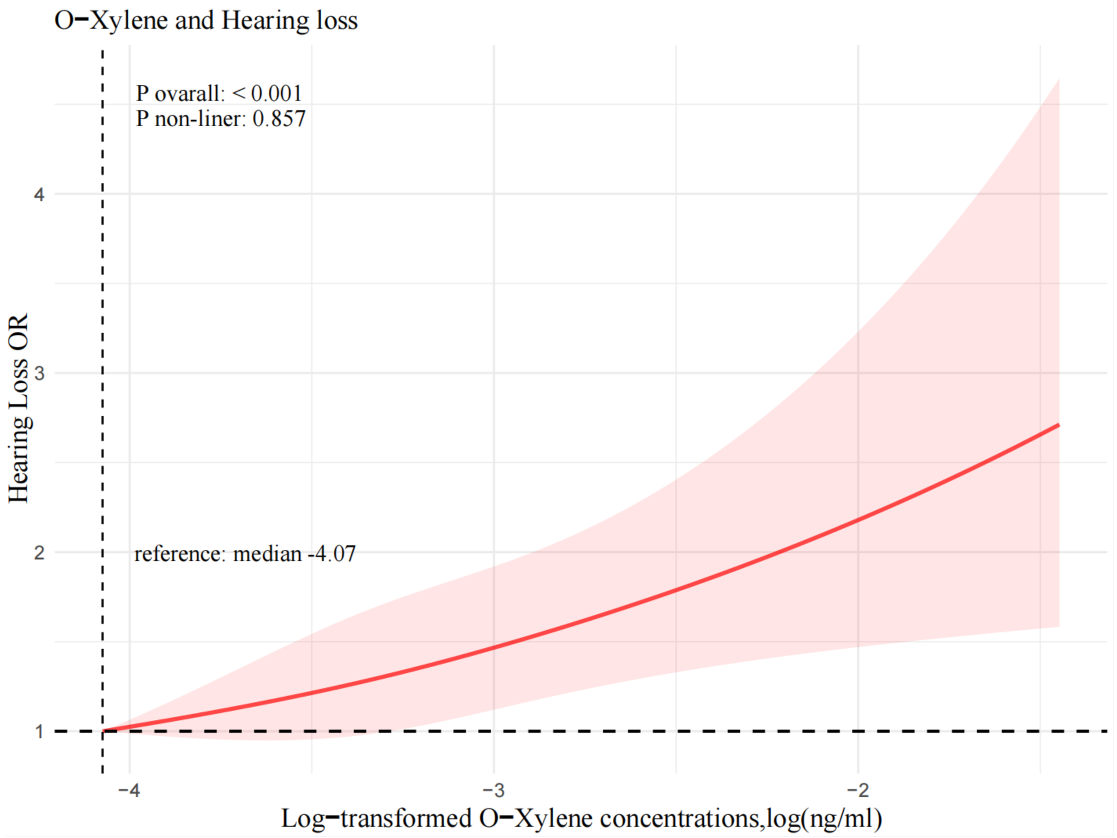

Supplementary Figure 6. The cubic spline of the association between log-transformed volume-based O-Xylene and risk of hearing loss. Models were adjusted for age (continuous, years), sex (male and female), BMI (continuous, kg/m^2), education levels (less than high school, high school and college or higher), race/ethnicity (Mexican American, Other Hispanic, Non-Hispanic White Non-Hispanic Black, Other Race - Including Multi-Racial), marital status (married, widowed, divorced, separated, never married, living with partner), PIR (<1.3, 1.3-3.5, >3.5), smoking status (no and yes), drinking status(no and yes), diabetes (no and yes), hypertension (no and yes), work noise(no and yes), recreational noise(no and yes), firearm noise(no and yes), and year cycle (2003-2004, 2011-2012, and 2015-2016). Knots =3. Abbreviations: BMI, body mass index; PIR, family income-poverty ratio.

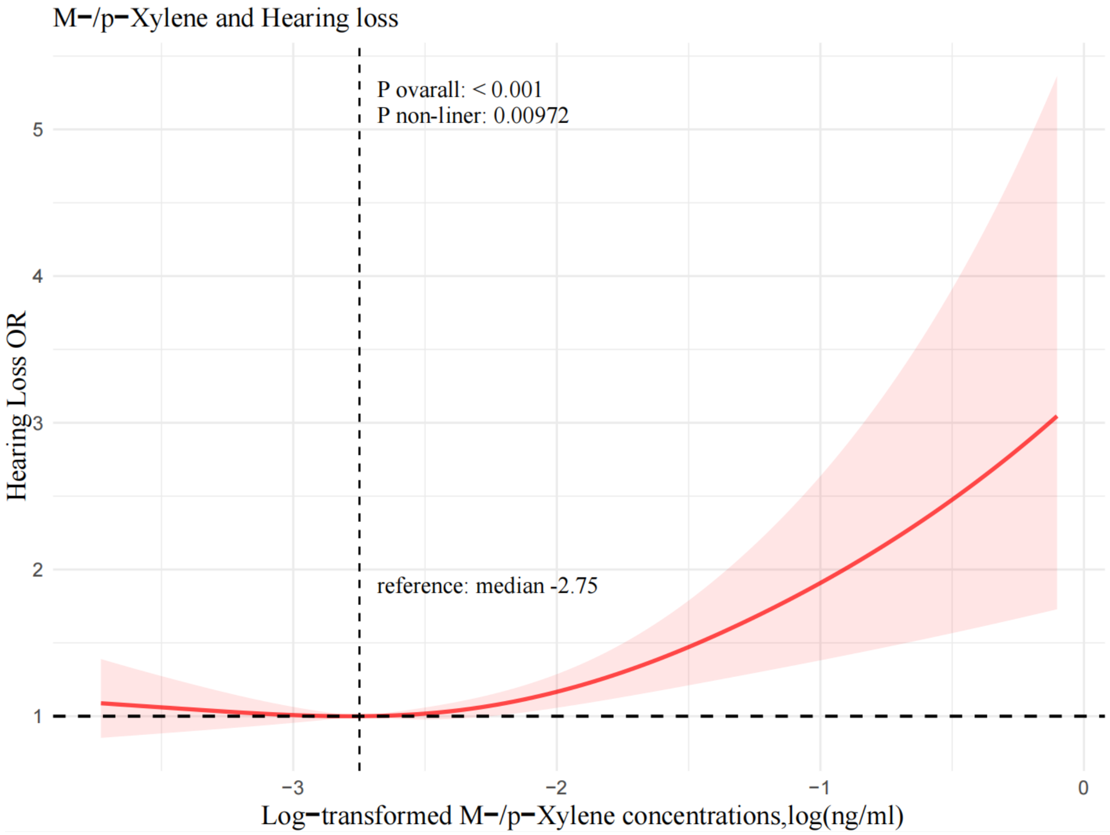

Supplementary Figure 7. The cubic spline of the association between log-transformed volume-based M-/P-Xylene and risk of hearing loss. Models were adjusted for age (continuous, years), sex (male and female), BMI (continuous, kg/m^2), education levels (less than high school, high school and college or higher), race/ethnicity (Mexican American, Other Hispanic, Non-Hispanic White Non-Hispanic Black, Other Race - Including Multi-Racial), marital status (married, widowed, divorced, separated, never married, living with partner), PIR (<1.3, 1.3-3.5, >3.5), smoking status (no and yes), drinking status(no and yes), diabetes (no and yes), hypertension (no and yes), work noise(no and yes), recreational noise(no and yes), firearm noise(no and yes), and year cycle (2003-2004, 2011-2012, and 2015-2016). Knots =3. Abbreviations: BMI, body mass index; PIR, family income-poverty ratio.

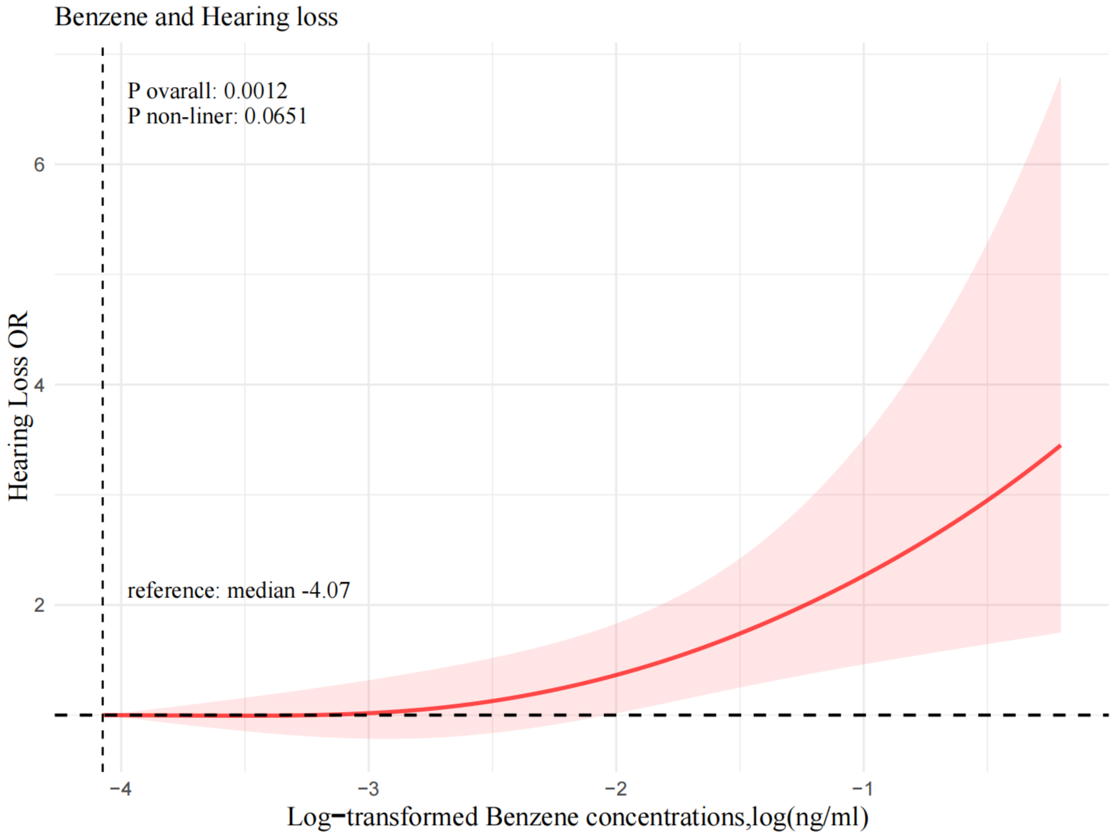

Supplementary Figure 8. The cubic spline of the association between log-transformed volume-based Benzene and risk of hearing loss. Models were adjusted for age (continuous, years), sex (male and female), BMI (continuous, kg/m^2), education levels (less than high school, high school and college or higher), race/ethnicity (Mexican American, Other Hispanic, Non-Hispanic White Non-Hispanic Black, Other Race - Including Multi-Racial), marital status (married, widowed, divorced, separated, never married, living with partner), PIR (<1.3, 1.3-3.5, >3.5), smoking status (no and yes), drinking status(no and yes), diabetes (no and yes), hypertension (no and yes), work noise(no and yes), recreational noise(no and yes), firearm noise(no and yes), and year cycle (2003-2004, 2011-2012, and 2015-2016). Knots =3. Abbreviations: BMI, body mass index; PIR, family income-poverty ratio.
